# Evaluating the Greenness of Wine Analytical Chemistry: A New Metric Approach

**DOI:** 10.3390/foods13223557

**Published:** 2024-11-07

**Authors:** Vasiliki Thanasi, Ana Beatriz Lopes, Paulo Barros, Natalia Ribeiro, Jorge M. Ricardo-da-Silva, Sofia Catarino

**Affiliations:** 1 LEAF-Linking Landscape Environment Agriculture and Food-Research Center, Instituto Superior de Agronomia, Universidade de Lisboa, Tapada da Ajuda, 1349-017 Lisboa, Portugal; vasilikithanasi@isa.ulisboa.pt (V.T.); anabeatrizglopes@hotmail.com (A.B.L.); jricardosil@isa.ulisboa.pt (J.M.R.-d.-S.); 2 Associate Laboratory TERRA, Instituto Superior de Agronomia, Universidade de Lisboa, Tapada da Ajuda, 1349-017 Lisboa, Portugal; 3 Instituto dos Vinhos do Douro e do Porto, I.P., Rua de Ferreira Borges, 27, 4050-253 Porto, Portugal; pbarros@ivdp.pt (P.B.); nribeiro@ivdp.pt (N.R.); 4 CeFEMA-Centre of Physics and Engineering of Advanced Materials, Instituto Superior Técnico, Universidade de Lisboa, Av. Rovisco Pais, 1, 1049-001 Lisboa, Portugal

**Keywords:** green analytical chemistry, greenness metric, wine analysis, sustainability

## Abstract

While the wine industry has already adopted some sustainable and environmentally friendly practices, special attention should be paid to the greenness of the analytical methodologies used. In this study, a new tool called “Green Wine Analytical Procedure Evaluation” (GWAPE) was developed to fulfill these “green” requirements. This framework offers a structured approach to evaluating the environmental impact of wine analysis processes, covering all the analytical steps from sample collection to the final results. GWAPE provides quantitative information, uses schematic representations, assigns varying levels of importance to green chemistry principles, and conducts detailed evaluations of hazard structures. Since wine analytical methods typically involve fewer highly hazardous reagents, certain criteria previously applied in green analytical chemistry should be integrated or omitted. In summary, GWAPE offers a customized and precise solution to help the laboratories of enology and the wineries assess their analytical methodologies’ environmental impact. As an example of application, the proposed metric was used to evaluate the greenness of three different standard analytical methodologies to determine sugars in wine, showing good discrimination ability.

## 1. Introduction

Wine is a complex matrix of organic acids, volatile species, polyphenols, polysaccharides and sugars, amino acids, other nitrogen substances, and inorganic species. Its composition depends on climatic, agricultural, and wine-making factors [1]. The quality control and authenticity assessment of wine is considered crucial to face the needs of the current globalization market. As a result, there is a growing need for appropriate analytical procedures to monitor wine production and assess wine quality and authenticity [2,3]. While detailed frameworks like LCA (Life Cycle Assessment) are already in use to assess the environmental impacts associated with all stages of a product’s life [4], special attention should be given to greening the analytical procedures [5]. To achieve this goal, the chemical society turned to green chemistry designs and solutions [6].

The International Organisation of Vine and Wine (OIV) has created a detailed compendium outlining the analytical methods used for wine and must analyses, recognized by governments worldwide [7]. In addition to organizing these methods, the OIV encourages scientific and technical innovation and promotes the sharing of information and knowledge to raise environmental sensitivity. By providing recommendations, international standards, and guidelines, the OIV aims to improve the productivity, safety, and quality of wine products and the conditions under which they are produced and marketed [7]. As a result, there is widespread emerging interest in the wine sector in harnessing analytical chemical innovation to meet sustainability criteria [5].

Green chemistry was first introduced in 1999 by Anastas enclosing the ‘design of chemical products and processes to reduce or eliminate the use and generation of hazardous substances’ [8]. Based on this definition, twelve principles have been established as guiding tools toward greener trends in chemistry [9]. Considering this approach was coined to implement green solutions related to organic synthesis and chemical engineering, these 12 “design rules” were not considered fully applicable to Analytical Green Chemistry. Based on these fundamental milestones the 12 principles were formulated to induce greenness in analytical methodologies [10]. However, for the successful management of green analytical chemistry, it is important to find a reliable way to quantify the level of greenness for each analytical alternative that claims to be greener.

Over time, different metric systems have been proposed for evaluating green analytical methodologies. The most popular options applicable in a wide range of analytical procedures include NEMI, Analytical Eco-Scale, Green Analytical Procedure Index (GAPI), and Analytical Greenness Calculator (AGREE) [11]. However, these evaluation tools have some advantages and disadvantages, concluding that an ideal metric does not exist [12]. More specifically, some of them, like NEMI, are characterized by a simple demonstration of the results but at the same time, there is a lack of quantitative evaluation [13]. Other tools, such as the Analytical Eco-Scale, only provide semi-quantitative results without demonstrating negative environmental impact schematically [14]. GAPI and AGREE can express results in an appealing and easy-to-understand way, but they may lack specific information about hazard structure when describing reagents [15,16]. However, the ability of these metrics to cover a wide range of analytical procedures, with good discriminative capacity, and more specifically their applicability in wine analytical procedures, is under study.

In previous preliminary work, the authors evaluated existing metrics after their implementation in general physicochemical wine analysis for a large number of parameters (free and total sulfur dioxide, total acidity, volatile acidity, chromatic characteristics, density, alcoholic strength, sugar content, wine turbidity, sulfates, malic acid, and yeast assimilable nitrogen) [12]. It became evident that some of the 12 green principles used as evaluation parameters were not entirely applicable to wine analysis. Notably, the first principle (selection of direct analytical techniques), the third (in situ measurements should be performed), and the sixth (derivatization should be avoided) consistently had similar values across procedures. This is because most analyses are conducted in laboratories rather than at production sites, and while sample preparation is often required for the wine matrix, derivatization reagents are usually unnecessary [12].

Furthermore, critical aspects for assessing greenness were missing or were not effectively addressed. While direct, multiparametric, and automated methods, such as FTIR, are advantageous in wine analysis, the calibration process supporting these measurements involves substantial energy and time, which is not reflected in the evaluation criteria. Additionally, the generation of consumable material waste during sampling and analysis, such as plastic bags and single-use plastic pipettes, needs to be considered. Time efficiency and energy usage, particularly in the storage of samples in temperature-regulated environments before analysis, are also overlooked by existing metrics [12].

Moreover, the discriminatory capacity of current metrics is questionable. For instance, AGREE failed to distinguish between traditional OIV methods (iodine titration) and other more automated approaches, such as potentiometry. In the case of the determination of Free Sulfur Dioxide, the OIV method (OIV-MA-AS323-04A, Type II Sulfur dioxide) scores a better greenness classification (0.55) in comparison with the potentiometric approach (0.52) [7]. In the determination of Total Sulfur Dioxide, the potentiometric approach is considered a more sustainable approach (0.50) in comparison with the OIV method (0.49) [6]. We note that the potentiometric option is semi-automated, and there is less contact with the reagents; however, the metric gives estimations with small differences [12].

Furthermore, there is a notable absence of adequate evaluation concerning hazardous reagents. Reagents’ environmental impact, user safety, and overall procedural safety are not effectively characterized. For example, the formol titration method for determining yeast assimilable nitrogen, considered one of the most hazardous in wine analysis, received a mediocre evaluation regarding reagent hazardousness but a good evaluation for operator safety. However, the evaluation overlooks the prolonged contact with the formaldehyde solution during the preparation of the formol solution and conditioning at pH 8, which poses carcinogenic risks [12].

Therefore, this study focuses on the development of a new tool for the evaluation of greenness, called GWAPE-Green Wine Analytical Procedure Evaluation. This evaluation approach is a structured framework for the evaluation of the environmental performance of an entire wine analytical methodology, from sample collection to final determination. At the same time, the proposed metric was created to compare different methodologies of wine analysis in terms of greenness.

## 2. Methodology

The new wine analysis evaluation tool named GWAPE was designed by reviewing and adjusting the most critical metrics to offer quantitative information. A comprehensive schematic representation like GAPI [15] and AGREE [16] was used, while the significance of hazard structures was highlighted in a detailed manner, similar to the Analytical Eco scale [13]. However, considering that wine analytical methods are not highly hazardous to the environment, there are few cases where there is a need to use toxic, hazardous reagents or derivatization agents. As a result, some of the GAC criteria were not considered (6th—derivatization should be avoided, and 10th—reagents from renewable sources should be preferred). At the same time, some new evaluation principles were introduced based on the needs of wine analytical chemistry, such as sample throughput and calibration. The storage of samples is separately considered under the first criterion for offline measurements. Criterion 6 requires an evaluation of energy consumption from all devices used, not just the primary analytical instrument. The 9th criterion includes consideration of material waste. The analytical methodology for evaluating greenness is based on 10 principles. The metric’s structure was analyzed in detail, including the representation of each principle and its importance for wine analytical procedures.

Based on the structure of the developed metric, specific software was developed using Python code to make this metric system friendlier to the users and facilitate its application (Supplementary Materials).

To test the applicability of the new metric in wine analytical procedures, the metric was used to evaluate the greenness of three analytical procedures of paramount importance: namely enzymatic determination of glucose and fructose [OIV-MA-AS311-02 Glucose and fructose, Type II method], determination of reducing substances [OIV-MA-AS311-01A Reducing substances, Type IV method] [7], and FTIR determination of glucose and fructose. Moreover, considering that these three analytical procedures are commonly used by the wine sector to evaluate the same parameter, the discriminatory capacity of the metric in terms of eco-friendliness was also assessed.

### 2.1. The Structure of Green Wine Analytical Procedure Evaluation (GWAPE)

**Figure 1:**
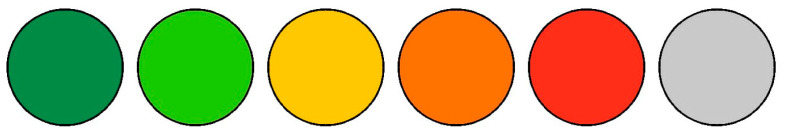
Colors of the evaluation scale: 5—dark green, 4—light green, 3—yellow, 2—orange, 1—red, no evaluation—grey.

**Figure 2:**
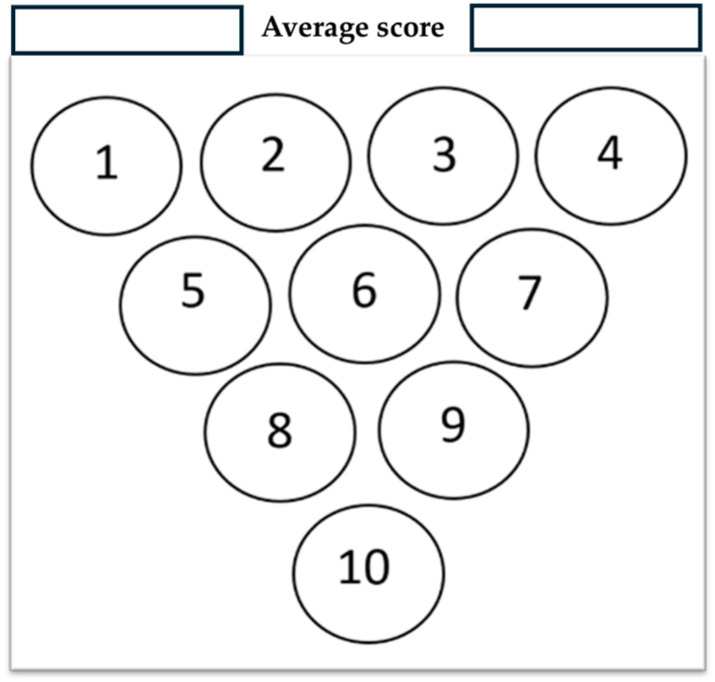
Visual summary of the metric GWAPE.

The GWAPE tool uses a circle resembling a grape berry to evaluate the level of greenness for each stage of an analytical procedure. It utilizes a color scale consisting of five levels to assess each stage, ranging from dark green (low), light green (medium-low), yellow (medium), orange (medium-high), and red (high), according to their environmental impact. Additionally, when the user chooses to exclude the evaluation of one parameter, the assessment of the berry will be colored grey (no evaluation) (Figure 1 and Figure 2).

To evaluate the eco-friendliness of wine analytical methods, the 12 principles of green analytical chemistry [9] were considered, and 10 new principles (1—Promotion of in situ measurements, 2—Integration of analytical processes and operations, 3—Selection of automated and miniaturized methods, 4—Choice of multi-analyte or multi-parameter methods, 5—Choose methods with a high sample throughput, 6—Minimization of the waste of energy, 7—Use of safer reagents, 8—Minimization of sample size, 9—Minimize Waste, 10—Calibration), with corresponding scores were created. These principles formed the basis for the evaluation, presented in the visual summary representing a grape (Figure 2). Each principle appearing as a single grape berry is rated on a scale of 1 to 5, with 1 (red color) indicating the least environmentally friendly analysis and 5 (dark green color) indicating the most ideal. An average score of 10 evaluations will appear at a bar on top of the grape representation, colored according to the average score (1–5) (Figure 2). This visual tool allows researchers to make judgments about conflicting green criteria, making it easier to compare wine analytical procedures.

**Figure 3:**
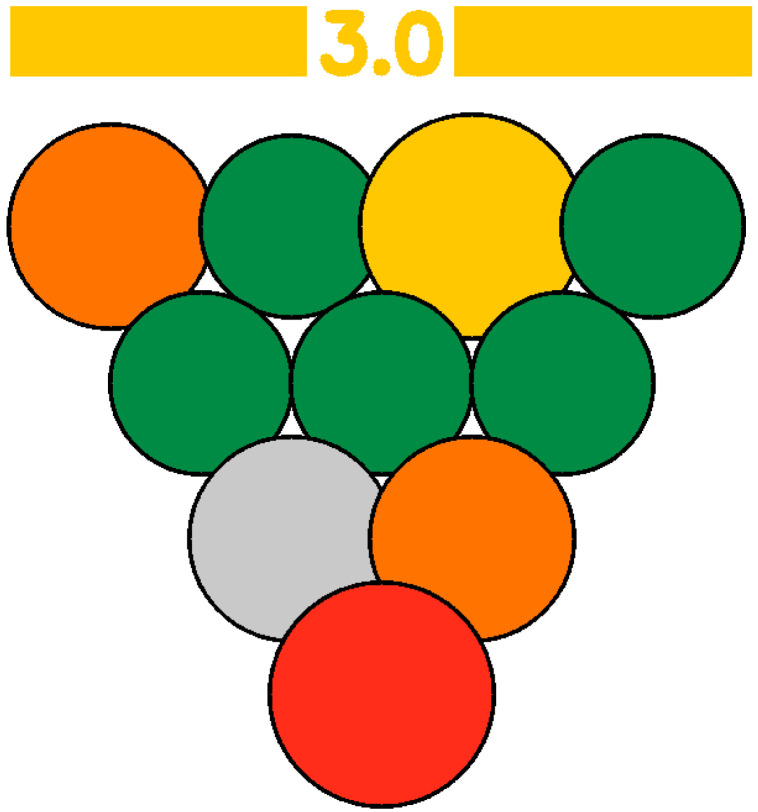
Example of the visual output of GWAPE according to different levels of weight attribution.

The final evaluation is defined by the numeric average and the corresponding color on the bar at the top of the grape in the visual output. Laboratories and wineries need to pay attention to each aspect separately, while also considering the overall result as a sum of the individual evaluations. The more principles that receive a green color, the more environmentally friendly the methodology is. At the same time, different weights may be assigned to each category based on its significance; external and winery laboratories may have different criteria for analytical procedures. Each user has the freedom to use a weight from 0 to 3 for each principle. The average of each principle now will be multiplied by its weight, and the size of the berry will be changed accordingly to demonstrate the change visually (higher weight leads to a bigger berry size, while for weight 0, the berry will be colored grey, indicating no evaluation). The final average score will now correspond to the weighted average (sum of each principle’s score multiplied by its weight/sum of the weights for all the principles). In an example demonstrated below, weight 0 was attributed to Principle 8—Sample volume, weight 1 was attributed to Principle 2—Number of unitary steps, Principle 4—Number of parameters under analysis, Principle 5—Sample throughput, Principle 6—Energy consumption, Principle 7—Hazard classification of reagents, weight 2 was attributed to Principle 1—Type of measurements, and Principle 11—consumable material waste and weight 3 to was attributed to Principle 3—Degree of automatization and miniaturization and Principle 10—Calibration (Figure 3).

The software developed to support the new metric is freely available to all users at the following link: https://alabe.pt/pt/sustentabilidade/sustentabilidade.php [Supplementary Materials, accessed on 19 October 2024]. Upon accessing this link, users will be redirected to a web platform with user-friendly interfaces that allow them to assign specific scores and weights for each of the 10 criteria. Additional supporting information on how to use the webpage can be found in Supplementary Materials.

### 2.2. The Evaluation Principles of GWAPE in Wine Analytical Procedures

#### 2.2.1. Promotion of In Situ Measurements

One important criterion, associated with the third principle of green analytical chemistry (CAG), stresses the significance of in situ sample preparation [10]. In the field of analytical wine chemistry, samples are typically derived from wine production, where continuous quality control is needed. This often requires additional time and energy for sampling [17]. For example, during the maturation control period of fharvest, berries are frequently sampled from various vineyard sites almost every day. These samples are then transported to wineries or external labs for analysis, and decisions regarding the appropriate harvest day are made accordingly. In some cases, samples are stored in temperature-regulated rooms or refrigerators, with preservatives added to prevent microorganism spoilage and oxidation (such as SO_2_) [18]. Therefore, it is important to consider and avoid waste of material, transportation, and storage energy whenever possible. To evaluate this criterion, according to the type of measurement and the location where it is taking place, an arithmetic scale of 1–5 is used (Table 1).

Measurements taken directly from the primary source are called in-line or in situ measurements. These measurements are analyzed in real-time, allowing for immediate observation of changes in the sample. For example, a light-emitting diode sensor can be used to measure color and total phenolics during red wine fermentation [19]. Online measurements are taken nearby and periodically, and feedback may be slightly delayed. An example of online monitoring of phenolic content is a contactless FT-NIR instrument [20]. On-site measurements involve transferring the sample preparation device to the sampling site. A device based on hyperspectral imaging can be used for in-field estimation of grape berry soluble solids and anthocyanin concentration [21]. Ex situ measurements involve sample preparation in the laboratory after sample collection and transportation. For example, collected grape berries are transported to the laboratory and stored at low temperatures in bottles or plastic bags before analysis. In this case, energy consumption during storage and the choice of storage material (e.g., plastic bags made of recycled material) should be given additional attention.

#### 2.2.2. Integration of Analytical Processes and Operations

In general, an analytical method is composed of multiple steps. Due to the complex nature of the wine matrix, a variety of individual steps are carried out during the chemical analysis of wine. Therefore, it is necessary to integrate these analytical steps to minimize material, energy, and time consumption according to the 4th principle of green analytical chemistry [22]. A unitary step refers to a single, indivisible action or stage within a process, often viewed as a fundamental operation that cannot be broken down further. To assess this factor, a numerical scale ranging from 1 to 5 was utilized, according to the steps of each procedure (Table 2).

A quick look at the enological procedures used in routine analyses shows that most involve, in most cases, fewer than 3 steps (sample preparation, calibration, and main analysis). However, sometimes the number of steps involved in a chemical process can be subjective. In certain cases, a complex process involving multiple reactions can be simplified into a single step. For instance, when determining assimilable nitrogen through formol titration, the calibration procedure for the formol solution is considered in the first step (preparation of the sample).

On the other hand, a more representative example of integration steps in wine analysis is the use of microfluidic devices. This lab-on-a-chip technology integrates wine analysis steps using a compact, portable system. The system usually consists of miniaturized channels to handle small volumes of fluid. A characteristic example is the quantification of phenolic compounds in wine samples with a microfluidic paper-based device [23].

#### 2.2.3. Selection of Automated and Miniaturized Methods

The 5th principle of green analytical chemistry emphasizes reducing the energy, solvents, and reagents required for analysis through the automation and miniaturization of methods. This approach also leads to lower risks for operators, as they are less exposed to chemicals. Miniaturized instrumentation is the scaled-down version of a larger laboratory-based analytical instrumentation. This version is compact and portable, performing similarly or equivalently to the full-sized version but with reduced size, weight, and power requirements (often using batteries) [24]. Automation refers to systems that employ technology to carry out tasks with minimal human intervention, typically by following a pre-programmed set of instructions or responding to specific conditions. An analytical instrument can include one or several functions that may be automated [25]. According to the level of human action needed during the analysis, the methods can be automatic, semi-automatic, non-automatic, or manual. For example, a reduced-scale approach is the quantification of volatile compounds by Headspace Solid-Phase Microextraction Gas Chromatography/Mass Spectrometry (HS-SPME-GC/MS) [26]. For the evaluation of this criterion, an arithmetic scale of 1–5 was considered (Table 3).

#### 2.2.4. Choice of Multi-Analyte or Multi-Parameter Methods

When conducting routine wine analysis, it is important to consider the various parameters that need to be measured, such as total acidity, pH, SO_2_, glucose, fructose, and ethanol. To streamline the process and reduce environmental impact, it is recommended to use multi-analyte or multi-parameter methods. These methods allow for the simultaneous analysis of multiple parameters, requiring fewer reagents, less time, and less energy. In monitoring applications, multi-analyte methods can detect issues early and enable proactive intervention and prevention. This fact saves time and avoids further use of reagents and energy. An example of a commonly used method for monitoring various parameters in wine production is Fourier transform infrared spectroscopy (FTIR) [27]. FTIR is utilized by winemakers and quality control laboratories to effectively evaluate different aspects of wine composition and quality. This method provides a comprehensive understanding of the wine’s characteristics, which enables better quality control, consistency, and adjustments in the winemaking process [28]. For the evaluation of this criterion, an arithmetic scale of 1–5 was considered (Table 4).

#### 2.2.5. Choose Methods with a High Sample Throughput

The time of the analysis (referring to instrument analysis and not including sample preparation time) is also important. When an analytical process is time-consuming, this translates to an increase in energy waste, especially when it involves instrument operation, and electricity and gas consumption increases while the analysis demands more time. To evaluate time concerning analysis, the throughput time formula can be used [Equation (1)].

TH = I/T,(1)

TH: Throughput of the sample for a given period (number of samples per hour), I: Inventory, number of samples when completing the analysis, T: Time to create the inventory in hours (hr).

To achieve high throughput, investing in instrumentation and method development is necessary. However, the cost per sample analyzed is significantly lower due to the reduction of manual labor. Additionally, this approach protects analysts from operational risks and chemical exposure. To simplify sample handling and speed up analysis time, techniques such as parallel manipulation of multiple samples and reduction of waiting times are implemented [29]. One example of a high-throughput method in wine analysis is automated spectrophotometric UV-visible spectroscopy [30]. Quality control is crucial in the wine industry, especially during vinification—a complex procedure that includes several steps and needs constant monitoring of various parameters at the same time. Usually, the number of samples to be analyzed increases significantly during harvest and fermentation periods. As a result, time and throughput are important factors for evaluating greenness and are considered as a separate criterion here. For the evaluation of this criterion, an arithmetic scale of 1–5 was considered (Table 5).

#### 2.2.6. Minimization of the Waste of Energy

It is important to note that while automated instruments are used to replace the analytical procedures of wet chemistry, it is crucial to pay attention to the amount of energy consumed. High electricity consumption leads to an increase in carbon dioxide emissions, which are a major contributor to greenhouse effects and global warming [31]. As a result, when assessing greenness, it is important to consider energy consumption. To evaluate the impact of this criterion, the total energy needed for the analysis of a sample using an electrical appliance or instrument should be evaluated. This number is then divided by the number of samples extracted to express energy consumption in watt-hours (Wh) per sample [32].

Therefore, it is important to consider any energy waste during analytical methodologies, whether it comes directly from the primary instrument used for the analysis (e.g., GC-MS or FTIR) or from other instruments used for sample preparation, such as centrifuges, ultrasonic baths, and evaporation devices. For the measurement of energy consumption, the information provided by the manufacturer of each instrument should be considered. It should be noted that this information only considers maximum energy consumption. For a more detailed approach, it is recommended to measure the total power output of instruments while in use [33]. For the evaluation of this criterion, an arithmetic scale of 1–5 was assigned (Table 6):

#### 2.2.7. Use of Safer Reagents

Considering the complexity of the wine matrix, in some cases, it is not possible to avoid the use of reagents. As a result, some of the analytical methodologies for wine analysis contain steps that require the use of reagents. A step toward safer analytical procedures would be the replacement of toxic reagents with safer alternatives and the harnessing of analytical wastes. Online decontamination can achieve this task (recycling, degradation, and passivation) [34]. However, their evaluation is important in terms of safety for both the environment and the operator. For the evaluation of this criterion, it is important to take into account the hazard pictograms implemented by the international Globally Harmonized System of Classification and Labelling of Chemicals (GHS). For example, sucrose does not have any hazardous pictograms. An iodine solution (0.05 mol I_2_/I–0.1 N) is labeled with one hazardous pictogram (hazardous to health) with an indication warning. Hydrochloric acid is labeled with 2 hazardous pictograms (hazardous and corrosive) with an indication of danger. Ethanol is characterized by 3 hazardous pictograms (flammable, harmful, and hazardous to health) with an indication of danger. According to the number of hazardous pictograms for each reagent used in a specific methodology, an arithmetic scale of 1–5 was assigned (Table 7):

#### 2.2.8. Minimization of Sample Size

The size of the sample is directly connected with the amount of waste generated and can indirectly impact, in some cases, the time and energy needed for the analysis (during heating, cooling, mineralizing), consumption of chemicals, and waste generation. At the same time, a smaller sample size is easier to combine with automation and portability [32]. Additionally, considering the commercial value of the wine samples as a product, especially in cases of aged high-priced wines, it is crucial to use a small volume of sample. According to the volume needed for the analysis, an arithmetic scale of 1–5 was attributed (Table 8).

#### 2.2.9. Minimize Waste

The main environmental threat in analytical chemistry is hazardous waste, which includes reagents. Handling, recycling, storing, and disposing of this waste require additional actions that consume more energy and time. Depending on the method of sample preparation, different amounts of waste depend on the nature of the sample, the chemicals, and the procedure involved [35]. When dealing with liquid samples like wine, the addition of reagents such as acids, bases, or salts makes the entire sample considered as waste. The cases where the sample does not contribute to waste are when no chemicals are added to the sample or when the sample does not come in contact with the reagents (e.g., headspace methods). All materials used for analysis are considered waste in this field, as the only output is the analytical result. Consumable materials such as cartridges, sorbents, filters, and single-use storage materials require special attention. An arithmetic scale of 1–5 is used to measure the production of analytical waste (Table 9).

#### 2.2.10. Calibration

Many of the factors being evaluated are shifting towards automated, multiparametric analytical methods. However, these methods typically require a calibration step that is considered essential but also consumes energy and time, and generates waste, including the use of calibration solutions, internal or external standards, standard reference materials, or isotope dilution. This aspect of analytical procedures is sometimes overlooked when evaluating the environmental sustainability of analytical methodologies. Nevertheless, it is essential not to underestimate the impact of these steps on waste generation [15]. For instance, FTIR measurements rely on extensive databases to construct mathematical models for the measurement of the factors being studied. Calibration is rated on a scale of 1–5 depending on the type of calibration being used (Table 10).

## 3. Results and Discussion

### 3.1. Application of GWAPE: Determination of Sugars in the Wine Case Study

GWAPE was applied to compare three different methods for the determination of sugars. The determination of sugars is a part of the routine analysis performed by wineries and external laboratories, especially during the vinification process. This determination in wine samples is crucial for three reasons: Firstly, it determines if all the fermentable sugar has been used up and if further fermentation is unlikely. Secondly, it indicates the sugar available to influence the sweetness of the wine. Finally, it is used to categorize wines as ‘dry’, ‘semi-dry’, or ‘sweet’ under specific regulatory environments. It is important to perform the sugar analysis methods according to these three objectives for effective wine production. Wine yeast can only metabolize glucose and fructose. Enzymes produced by yeast hydrolyze sucrose into glucose and fructose, leaving only trace amounts in the finished wine. Pentose sugars such as arabinose, xylose, ribose, and rhamnose remain in wine at levels of 0.4 to 2 g/L. The most significant sugars in wine are unfermented glucose and fructose, and small amounts of pentoses [36].

Three methods for determining sugar levels in wine, namely the enzymatic determination of glucose and fructose [OIV-MA-AS311-02 Glucose, and fructose, Type II method], determination of reducing substances [OIV-MA-AS311-01A Reducing substances, Type IV method], and FTIR determination of glucose and fructose, were compared using the green metric GWAPE to evaluate their environmental friendliness [7,28]. According to the schematic output, the greenest turned out to be the FTIR method, reaching the best average score (3.5-light green with five berries colored dark green, one yellow, three orange, and one red). The method had the worst evaluation for the 10th principle concerning the calibration, as expected. The second greenest was considered the enzymatic approach (average score 2.5-yellow with two berries colored dark green, one light green, one yellow, two orange, and four red). The method had the worst evaluation for the 4th (choice of multi-analyte or multi-parameter methods), 5th (choose methods with a high sample throughput), 7th (use of safer reagents), and 9th (minimize waste) principles. The least green performance gave the method for the determination of reducing substances (average score 1.8-orange with only one berry colored dark green, one light green, one dark yellow, and seven orange). The method had the worst evaluation for the 3rd (integration of analytical processes and operations), 4th (choice of multi-analyte or multi-parameter methods), 5th (choose methods with a high sample throughput), 6th (minimization of the waste of energy), 7th (use of safer reagents), 8th (minimization of sample size), and 9th (minimize waste) principles. For a more straightforward comparison of the three evaluations, each principle was assigned an equal weight of 1. A more detailed evaluation, along with the average scores and the schematic outputs, is demonstrated below (Table 11).

#### 3.1.1. Analysis of Glucose and Fructose (Enzymatic Method)

Glucose and fructose may be determined individually by an enzymatic method, with the sole aim of calculating the glucose/fructose ratio. This analysis is based on the capability of enzymes such as hexokinase, glucose-6-phosphate dehydrogenase, and phosphoglucose isomerase to catalyze reactions that involve glucose and fructose as substrates. The resulting coenzyme, NADPH (nicotinamide adenine dinucleotide phosphate), which is produced from redox reactions, is measured using UV-VIS spectrophotometry at 340 nm (OIV-MA-AS311-02 Glucose, and fructose, Type II method) [7].

This measurement was conducted “Ex situ without storage” at a temperature of 20–25 °C, eliminating the need for a refrigerator. The process consists of two unitary steps: sample preparation and main determination. During sample preparation, the sample is diluted with water based on the estimated amount of glucose + fructose per liter (g/L). In the main determination, a spectrophotometer adjusted to a 340 nm wavelength is used to take measurements with various enzymatic solutions. This semi-automatic and non-miniaturized method analyzes only one parameter. One hour is required to analyze a single sample (with a sample throughput of 1). The spectrophotometer used has an energy consumption of 0.008 kWh, based on the manufacturer’s specifications. However, this method involves the use of at least one hazardous reagent with an indication of danger (sodium hydroxide). A sample volume of 0.2 mL is required, while the liquid waste generated is 14 mL. The analysis also generates consumable material waste (such as tips for electronic pipettes and paper). The method does not require a calibration process.

#### 3.1.2. Determination of Reducing Substances

When measuring sugars that contain ketonic and aldehydic functional groups, reducing substances are used. These are measured by their ability to reduce an alkaline solution of a copper salt. It is important that the sugar content of the liquid in which sugar is to be determined lies between 0.5 and 5 g/L. When clarifying dry wines, they should not be diluted. However, sweet wines should be diluted during clarification to bring the sugar level within the limits. The wine is treated with zinc ferrocyanide (II) in this procedure. In an alkaline environment, Cu^2+^ ions oxidize the carbonyl groups of the reducing sugars. Cupric ions are present in excess relative to the amount of reducing sugars. The excess of cupric ions is then evaluated using potassium iodide (OIV-MA-AS311-01A Reducing substances, Type IV method) [7].

This evaluation method is categorized as “Ex situ without storage” and involves two unitary steps. It is a manual and non-miniaturized method that can determine one parameter in each analysis with a sample throughput of 0.75. A hot plate evaporator was used, consuming 3.75 kWh of energy. During the analysis, reagents with more than three hazardous pictograms and warning indications were used. The sample volume used in this method is 50 mL, resulting in a liquid waste of 380 mL and creating consumable material waste during the determination. It is important to note that this method does not require calibration.

#### 3.1.3. Determination of Glucose and Fructose by FTIR

The IR techniques work by measuring how molecules absorb IR radiation after changing their vibrational and rotational modes due to energy absorption. Each molecule contains different functional groups, such as carbonyl or amide groups, that have a unique IR absorption at specific frequency ranges. When exposed to IR radiation, these functional groups vibrate and produce a characteristic absorption signal [28].

This method is considered ex situ without storage, while taking place with one unitary step. It is an automatic and non-miniaturized technique that can analyze more than five parameters at the same time with a sample throughput of 144. The energy consumption of the equipment is less than 0.1 kWh, as indicated by the manufacturer, and it does not use any hazardous reagents during the analysis. The method does not generate any consumable material waste after the analysis, and the liquid waste generated is only 5 mL, which is the same volume as the sample used. However, the calibration process for this method is extended and requires regular readjustment, which results in volume calibration waste that exceeds 10 mL.

## 4. Conclusions

According to the rising interest in green analytical chemistry (GAC), there is a growing need for comprehensive tools to evaluate and compare analytical methods concerning their environmental effects. While the basic principles of GAC are simple and clear, they may not be sufficient to thoroughly assess enological analytical procedures. Some existing assessment tools may overlook important aspects such as calibration, or others focus on factors that are not applicable in wine analytical chemistry, such as derivatization.

To address these limitations, a new tool called GWAPE (Green Wine Analytical Procedure Evaluation) was introduced. GWAPE is a tool designed to offer a comprehensive evaluation of wine analytical procedures, by assessing various components, including reagents, instruments, transport and storage, occupational hazards, waste generation, sample throughput, and calibration. GWAPE assigns scores to each component in a procedure and calculates a total score for that method. This scoring system allows for a straightforward comparison of the environmental sustainability of different wine analytical methods from sample collection to the final determination.

Highlighting unique considerations applicable to wine analytical methods, some of the Green Analytical Chemistry (GAC) criteria were either combined or left out, and new criteria were introduced. These fresh criteria encompass aspects such as calibration requirements and the time taken for analysis, expressed as the sample throughput. Criterion 6 shifts the focus to evaluating the energy consumption not only of the primary analytical instrument but also of all the devices in use. The first criterion also considers sample storage, especially when dealing with offline measurements. Additionally, the ninth criterion introduces the consideration of material waste in the evaluation process. Each of these criteria is assessed on a scale ranging from 1 (indicating the least environmentally friendly analysis) to 5 (representing an ideal, environmentally friendly analysis). Furthermore, assigning different importance weights to each category, depending on their significance, was proposed. For example, external laboratories and winery laboratories may have distinct sets of criteria for evaluating analytical procedures, and a scale of 0–3 is proposed for this purpose.

While the proposed metric was used in this study to evaluate the greenness of three methodologies for the determination of sugars in wine as an example of its application, it was developed to be applied in different analytical procedures for the determination of all parameters related to wine analysis. At the same time, the metric was created to perform a comparison of different wine analytical methodologies in terms of their greenness. The metric was developed based on the needs of wine analysis; therefore, it focuses mainly on wine analytical determinations. However, keeping in mind that the evaluation is based on the principles of green analytical chemistry, it could provide a general overview in terms of eco-friendliness for other analytical determinations and other foods and beverages (e.g., beer, wine spirits).

Moreover, this evaluation relates to the green character of wine analysis and does not evaluate the accuracy and precision of the methodologies. Additionally, neither the cost of the methods nor the equipment was analyzed in detail. Only in some cases could it be related to the time and energy needed for each analysis. In cases of methodologies involving analytical instruments (e.g., FTIR), the evaluation of greenness is related to operational use. However, it is important to acknowledge that the environmental impact associated with the production of such devices is not considered by the metric. As a result, each laboratory, winery, and individual should consider all of the above when selecting the most environmentally friendly approach for their specific needs.

In conclusion, OIV, as an official organization, is already contributing to the international harmonization of existing practices and standards and at the same time is promoting sustainability. Bearing in mind that there is no official evaluation technique for the green character of the analytical wine methodologies, establishing a quantification metric tool is of great importance.

## Figures and Tables

**Table 1:** Scores of each type of measurement based on its location.

Type of Measurement	Score
In-line/In situ	5
Online/In situ	4
On-site	3
Ex situ without storage	2
Ex situ with storage	1

**Table 2:** Attribution of scores according to the number of unitary steps in analytical procedures.

Number of Unitary Steps	Score
1	5
2	4
3	3
4	2
≥5	1

**Table 3:** Attribution of scores according to degree of automation and miniaturization.

Degree of Automatization and Miniaturization	Score
Automatic-miniaturized	5
Semi-automatic-miniaturized	4
Non-automatic-miniaturized	3
Automatic, non-miniaturized	3
Semi-automatic, non-miniaturized	2
Manual, non-miniaturized	1

**Table 4:** Attribution of scores according to the number of parameters under analysis in one single run.

Number of Parameters Under Analysis	Score
≥5 parameters at one single run	5
≥2 parameters in one single run	3
One parameter in one single run	1

**Table 5:** Attribution of scores according to the sample throughput.

Sample Throughput (TH)	Score
ΤH > 140	5
120 < TH ≤ 140	4
100 < TH ≤ 120	3
80 < TH ≤ 100	2
ΤH ≤ 80	1

**Table 6:** Attribution of scores according to the level of energy consumption during the analysis.

Energy Consumption	Score
≤0.1 kWh per sample	5
≤1.5 kWh per sample	3
>1.5 kWh per sample	1

**Table 7:** Attribution of scores according to hazard classification of the reagents used.

Hazard Classification of Reagents	Score
No hazardous pictograms	5
1 hazardous pictogram with indication warning	3
2 hazardous pictograms with indication warning	2
≥3 hazardous pictograms with indication warning or at least 1 hazardous pictogram with indication danger	1

**Table 8:** Attribution of scores according to the sample volume used.

Sample Volume (V_s_)	Score
V_s_ < 0.1 (mL)	5
0.1 ≤ V_s_ ≤ 1 (mL)	3
1 < V_s_ ≤ 10 (mL)	2
V_s_ > 10 (mL)	1

**Table 9:** Attribution of scores according to the volume and type of waste produced after the analysis.

Analytical Waste	Score
Liquid waste < 1 mL and no consumable material waste	5
Liquid waste and < 1 mL and consumable material waste	4
Liquid waste 1–10 mL and no consumable material waste	3
Liquid waste 1–10 mL and consumable material waste	2
Liquid waste > 10 mL and no consumable material waste	2
Liquid waste > 10 mL and consumable material waste	1

**Table 10:** Attribution of scores according to the type of calibration used.

Type of Calibration	Score
The analytical methodology does not contain a calibration step	5
The analytical methodology contains calibration steps with no production of analytical waste	4
The analytical methodology contains calibration steps with the production of analytical waste < 1 mL	3
The analytical methodology contains calibration steps with the production of analytical waste 1–10 mL	2
The analytical methodology contains calibration steps with the production of analytical waste > 10 mL	1

**Table 11:** Presentation of the scores for each green principle and the visual output after the application of GWAPE.

Green Principles	1st	2nd	3rd	4th	5th	6th	7th	8th	9th	10th	Visual Output
**Enzymatic determination**	Ex situ without storage	2 unitary steps	Semi-automatic, non-miniaturized	One parameter	1 sample/hour	0.008 kWh	1 hazardous reagent with indication danger	sample volume = 0.2 mL	liquid waste = 14 mL + material waste	No calibration	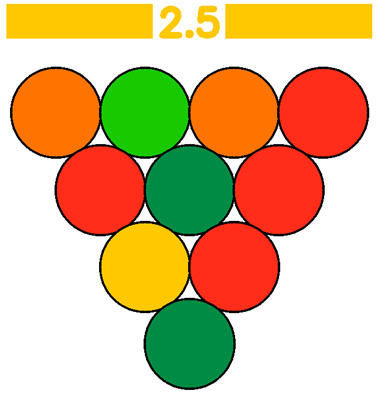
**Determination of reducing substances**	Ex situ without storage	2 unitary steps	manual, non-miniaturized	One parameter	0.75 samples/hour	3.75 kWh	>3 hazardous pictograms with warning	Sample volume = 150 mL	Liquid waste = 380 mL + material waste	No calibration	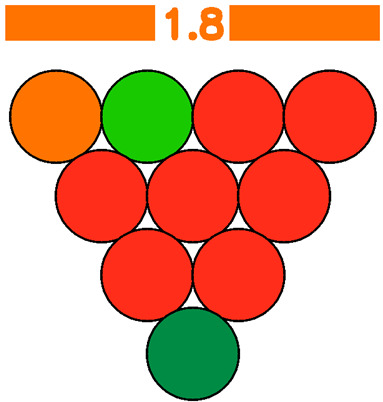
**FTIR** **determination of glucose and fructose**	Ex situ without storage	1 unitary step	Automatic, non-miniaturized	>5 parameters	144 samples/hour	0.1 kWh	None	Sample volume = 5 mL	Liquid waste = 5 mL + no material waste	Calibration needed	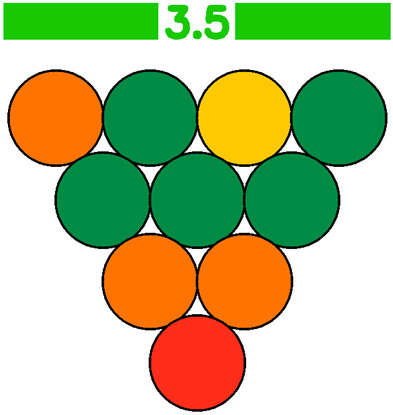

## Data Availability

The original contributions presented in this study are included in the article/Supplementary Materials. Further inquiries can be directed to the corresponding author.

## Supplementary Materials

The following supporting information can be downloaded at: https://www.mdpi.com/article/10.3390/foods13223557/s1, Development of software. To make this metric system more friendly to the users and facilitate its application, software was developed using Python code.
