# Supplementary material for: Evaluating the Greenness of Wine Analytical Chemistry: A New Metric Approach
Source: Foods. 2024 Nov 7;13(22):3557. doi: 10.3390/foods13223557 (PMC11592660; doi:10.3390/foods13223557)
Supplement: Supplementary file 1 [file foods-13-03557-s001.zip › Software Supporting Information for GWAPE.pdf]

Software Supporting Information for GWAPE

The GWAPE tool uses a circle resembling a grape berry to evaluate the level of greenness for each stage of an analytical procedure. It utilizes a color scale consisting of five levels to assess each stage. The tool displays a grape diagram with ten berries (Figure S1) to evaluate each step of the methodology, ranging from dark green (low), light green (medium-low), yellow (medium), orange (medium-high) to red (high) according to their environmental impact (Figure S2).

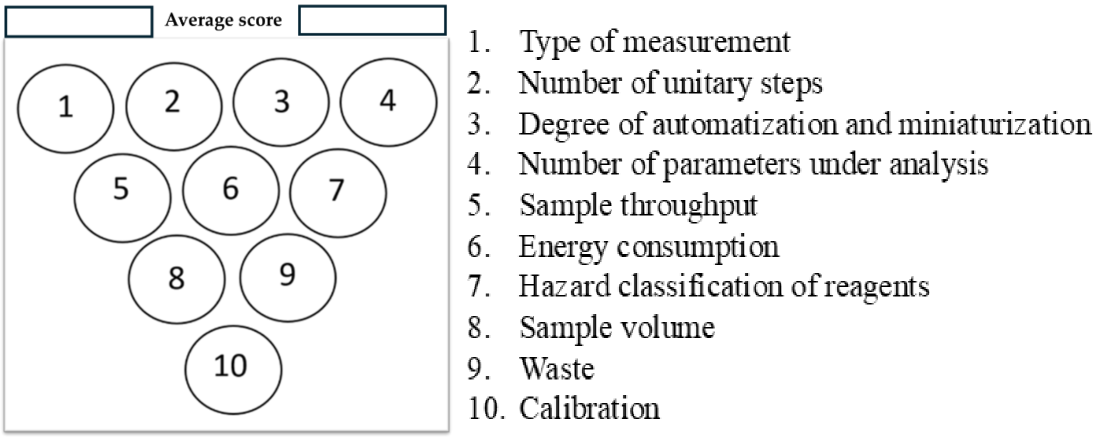

Figure S1: Visual summary of the metric GWAPE and the respective evaluation criteria 1-10

Each principle appearing as a single grape berry is rated on a scale of 1 to 5, with 1 indicating the least environmentally friendly analysis and 5 indicating the most ideal. This visual tool allows researchers to make their judgments about conflicting green criteria (1-10), making it easier to compare wine analytical procedures. At the same time, different weights may be assigned to each category based on their significance; external and winery laboratories may have different criteria for analytical procedures. Each user has the freedom to use a weight from 0 to 3 for each principal. The average of each principle now will be multiplied by its weight and the size of the berry will be changed accordingly to demonstrate the change visually (higher weight leads to a bigger berry size, while for weight 0 the berry will be colored grey indicating no evaluation).

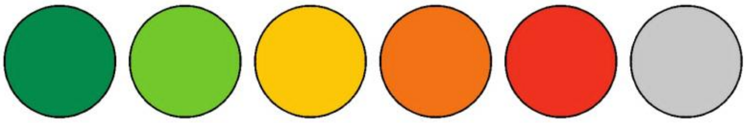

Figure S2: Colors of the evaluation scale: 5-dark green, 4-light green,3-yellow, 2-orange, 1-red, no evaluation-grey

To make this metric system more friendly to the users and facilitate its application, software was developed based on Python code. The use of the software is free to all users through the link:

https://alabe.pt/pt/sustentabilidade/sustentabilidade.php

Entering this link, the users will be redirected to a web environment, containing interfaces for assigning particular scores corresponding to each of the 10 criteria as it was analyzed in detail in the article, through the tab menu in the left corner of the main window, as it is demonstrated in Figure S3.

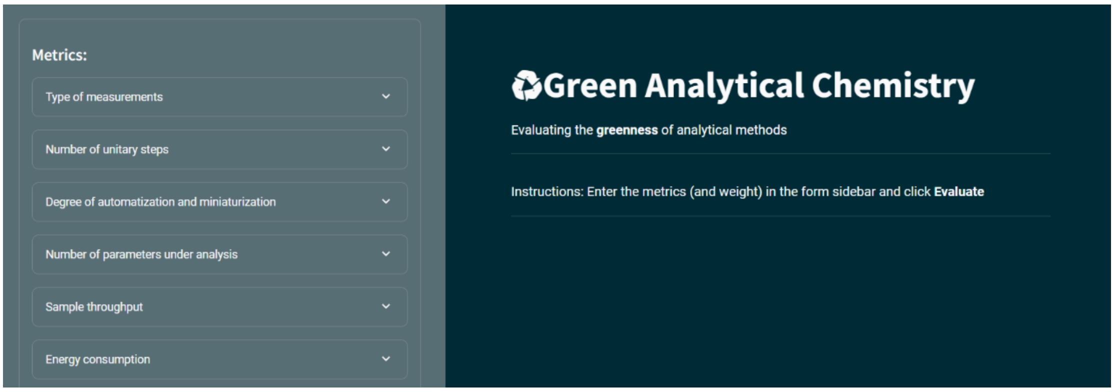

Figure S3: Webpage of GWAPE software

The analytical methodology for evaluating greenness is based on 10 principles. Each principle represents a different aspect and is assigned a number from 1 to 5. Additionally, the user can assign a specific weight from 0 to 3 according to his needs and the importance assigned to each principle.

The first principle stresses the significance of in situ sample preparation and the user can choose between **In-line/In-situ, On-line/In-situ, On-site, Ex-situ without storage, or Ex-situ with storage** according to the type of measurement that took place (Figure S4).

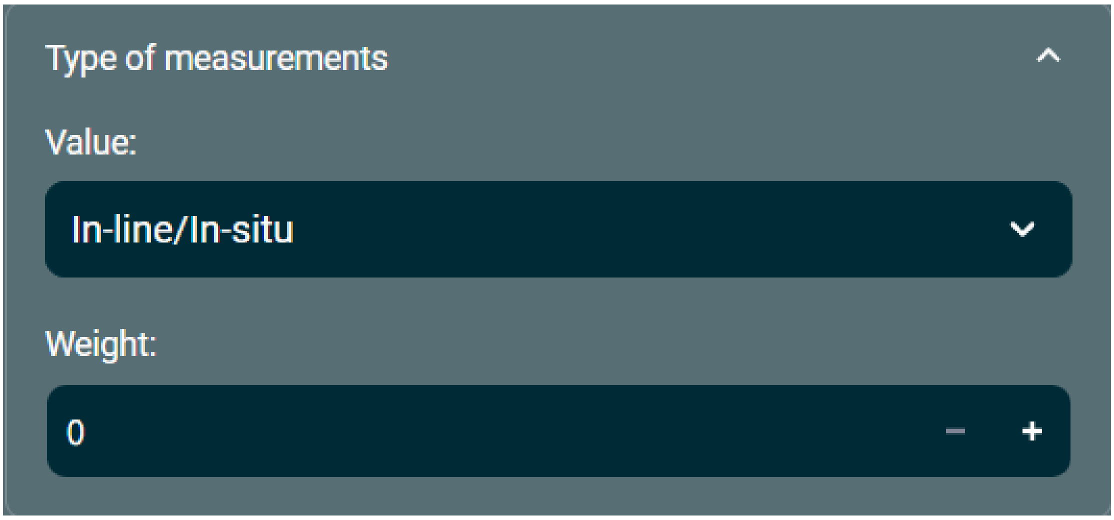

Figure S4: 1st interface considering the type of measurement

In the second interface, the user can input the number of steps in the analytical procedure to evaluate the degree of integration between analytical processes and operations (Figure S5).

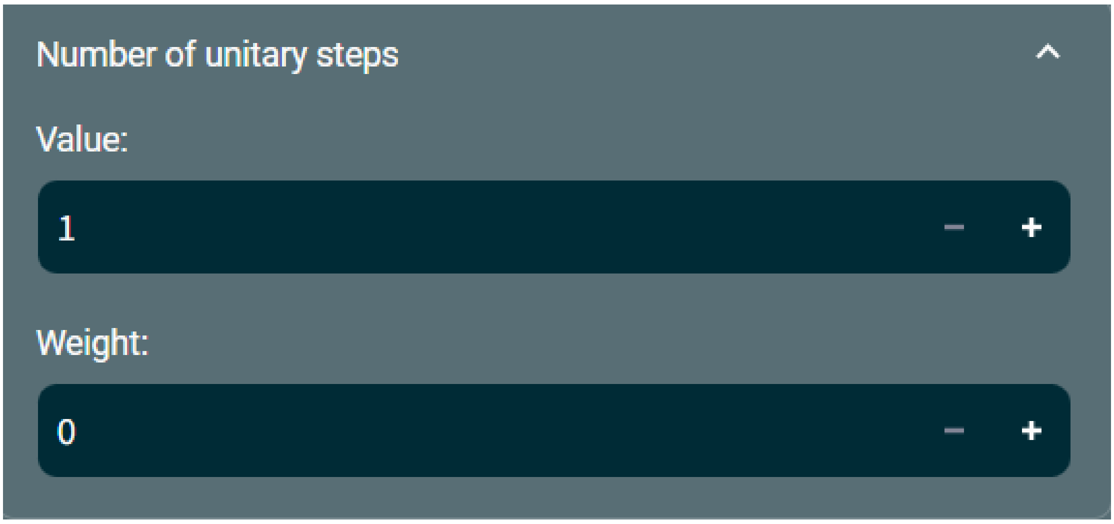

Figure S5: 2nd interface considering the number of unitary steps

The 3rd criterion of GWAPE emphasizes the reduction of energy, solvents, and reagents required for analysis through automation and miniaturization of methods and the user can choose between **Automatic-miniaturized, Semi-automatic-miniaturized, Non-automatic-miniaturized, Automatic, non-miniaturized, Semi-automatic, non-miniaturized, Manual, non-miniaturized** analytical procedure (Figure S6).

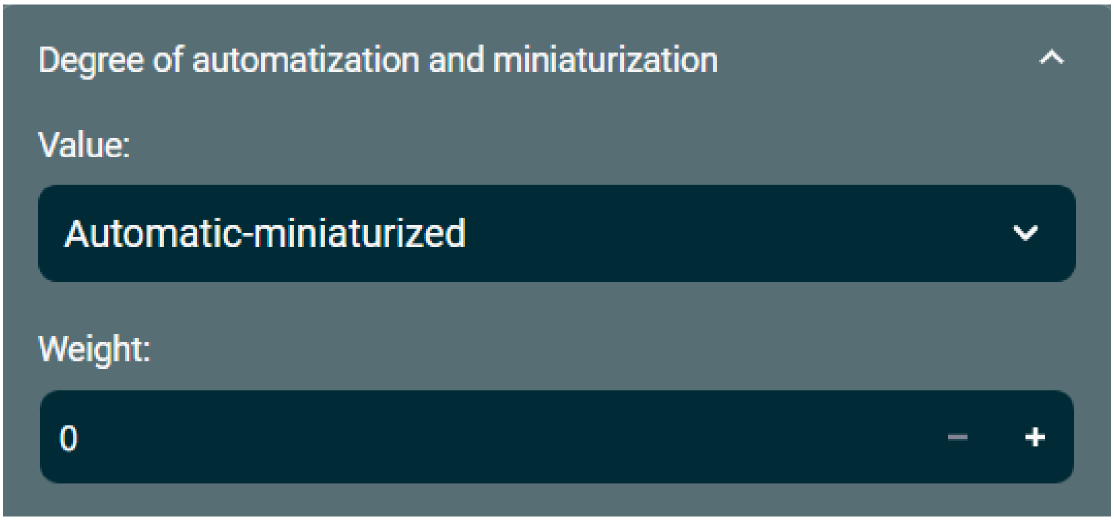

Figure S6: 3rd interface considering the degree of automatization and miniaturization

Through criterion 4, it is recommended to use multi-analyte or multi-parameter methods and the user has to indicate the number of parameters under analysis in a single run (Figure S7).

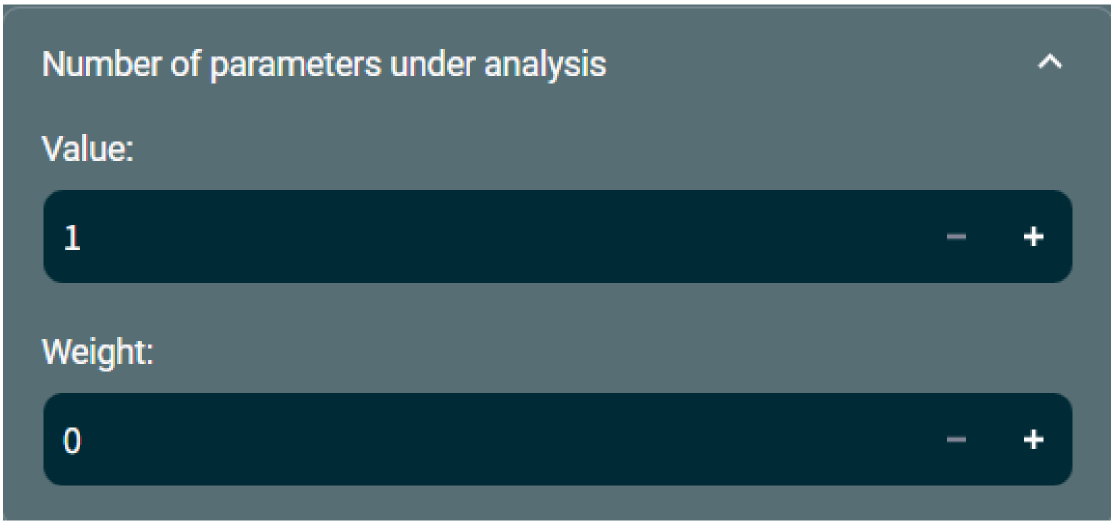

Figure S7: 4th interface considering the number of parameters under analysis

To evaluate the time concerning the analysis the formula of the throughput time formula was used in Criterion 5 and the user has to enter the value of analysis throughput (Figure S8) using the corresponding equation {1}

TH = I / T, {1}, were TH: Throughput of the sample for a given period, I: Inventory, number of samples when completing the analysis, T: Time to create the inventory in hours

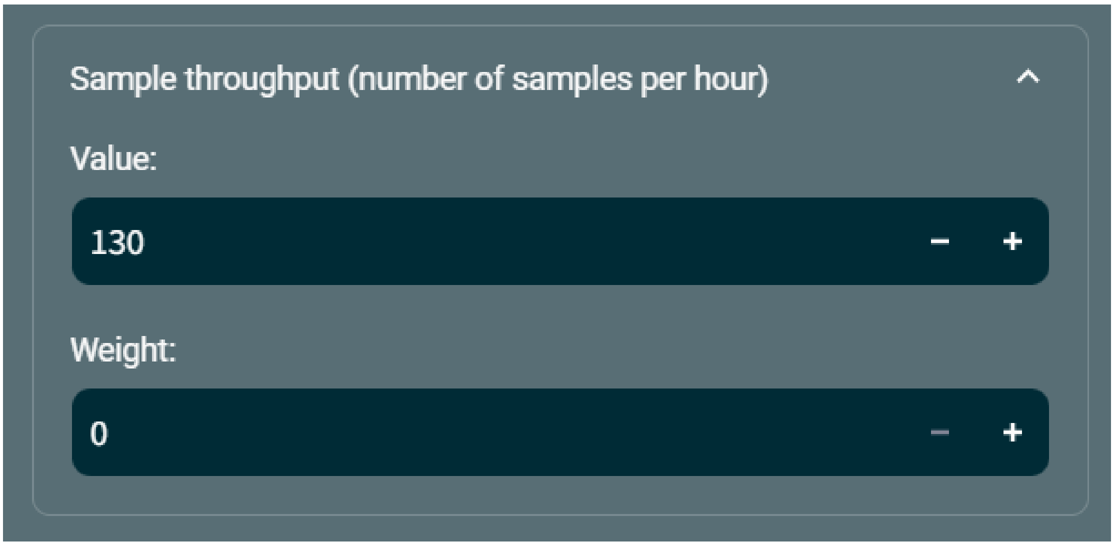

Figure S8: 5th interface considering the sample throughput of the analysis

Principle 6 concerns the energy consumption as a total power output of instruments while in use measured in KWh (Figure S9).

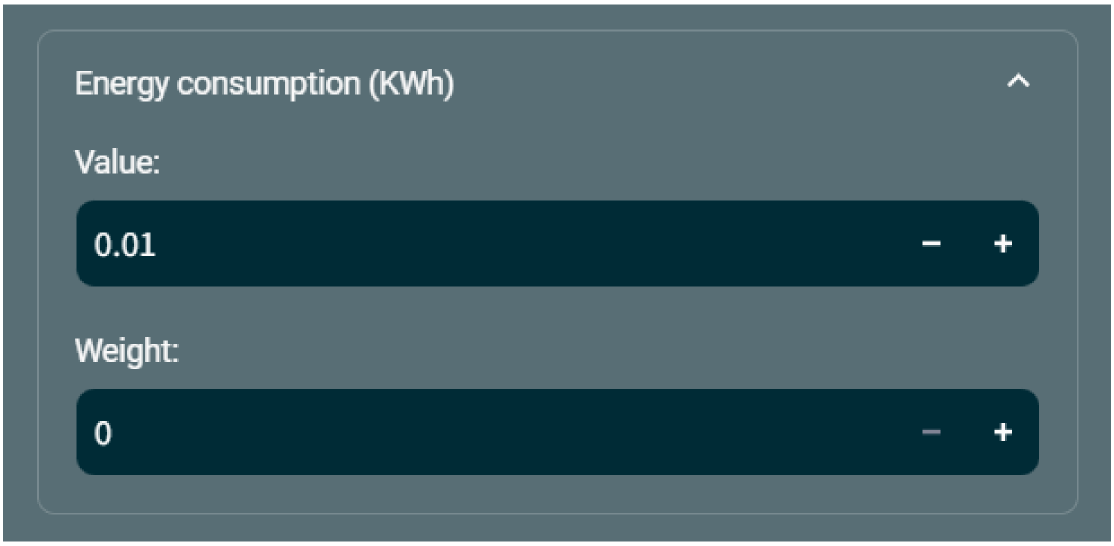

Figure S9: 6th interface considering the energy consumption

For the evaluation criterion 7, it is important to take into account the Hazard pictograms implemented by the international Globally Harmonized System of Classification and Labelling of Chemicals (GHS). According to the number of hazardous pictograms for each reagent used for a specific methodology, the user can pick out among the options: **No hazardous pictograms, 1 hazardous pictogram with indication warning, 2 hazardous pictograms with indication warning, ≥3 hazardous pictograms with indication warning or at least 1 hazardous pictogram with indication danger** (Figure S10).

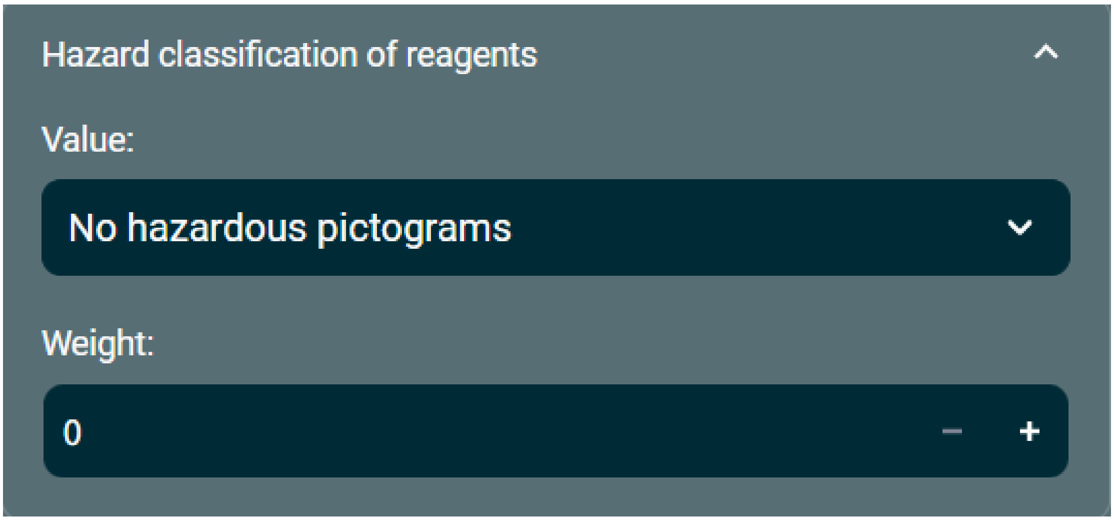

Figure S10: 7th interface considering the GHS hazard pictograms

Principle 8 evaluates the process according to the volume needed for the analysis, and the user can insert the volume of the sample in interface 8 (Figure S11).

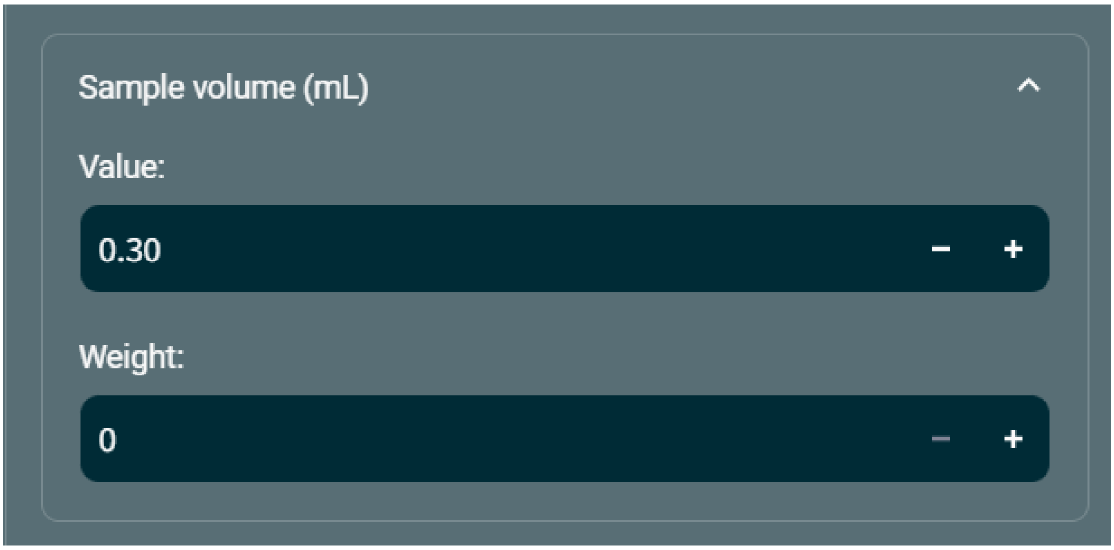

Figure S11: 8th interface considering the sample volume

Principle 9 analyzes the influence of waste depending on the waste volume but also the type of waste (reagents or consumable material). The user needs to indicate in the 9th interface if any consumable material was used and in the 10th interface the waste volume that was created during the analysis (Figure S12).

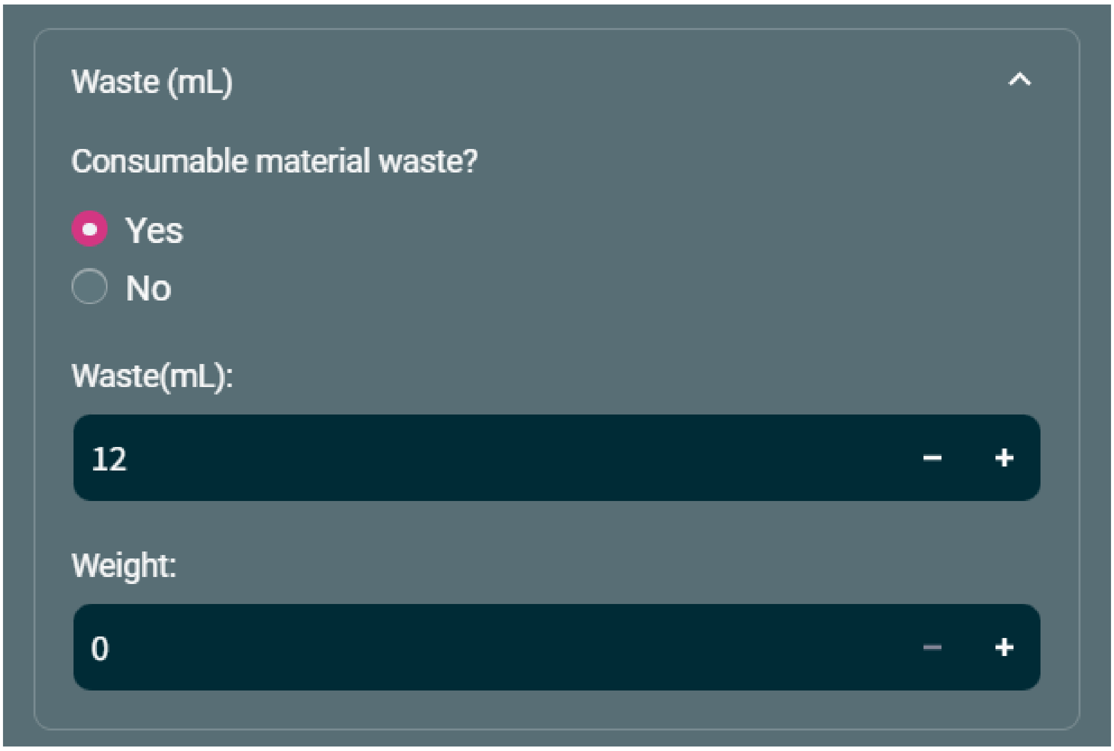

Figure S12: 9th interface considering the waste

In Criterion 10, the need for calibration is rated depending on the type of calibration being used. The user needs to indicate if the analysis is based on the calibration process in the 11th interface and which is the relative volume of waste produced during the calibration process in the 12th interface (Figure S13).

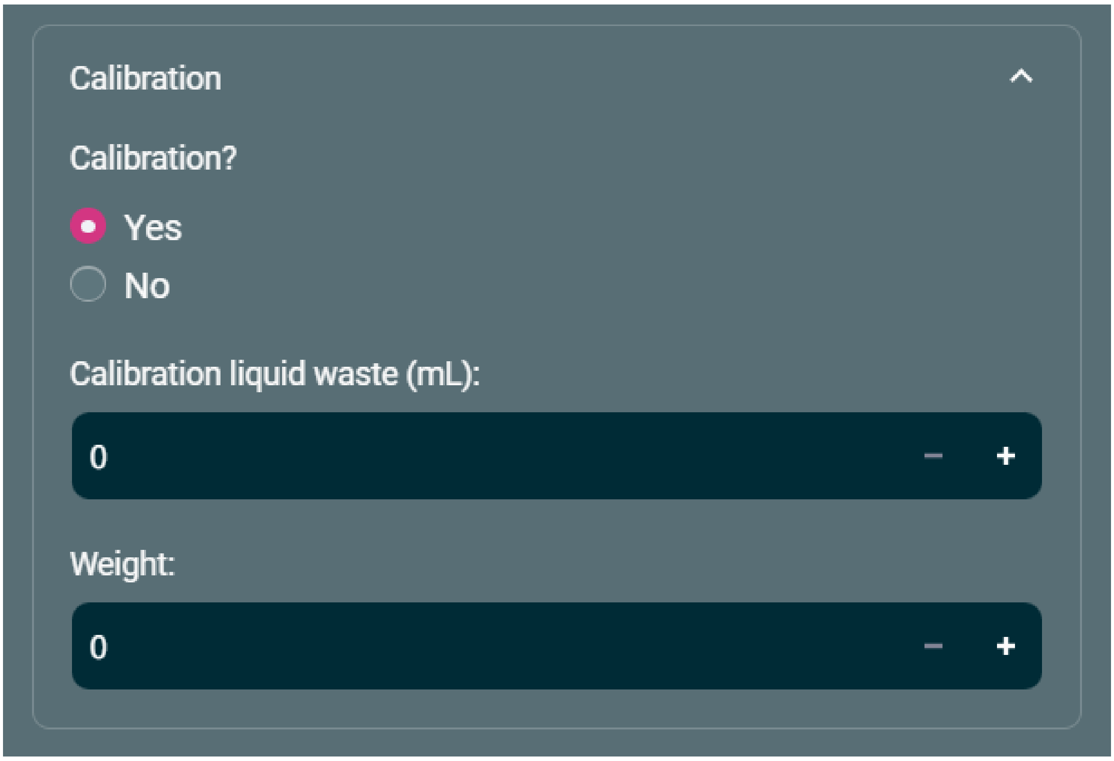

Figure S13: 10th interface considering the calibration

After the user fulfills all the necessary information in the 12 interfaces in the sidebar, needs to click **Evaluate** (Figure S14).

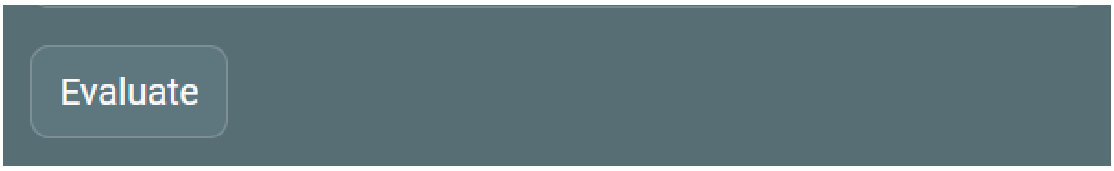

Figure S14: Evaluation bar

The final evaluation is represented by the colors in the visual output (Figure S15). Laboratories and wineries need to pay attention to each aspect separately, while also considering the overall result as a sum of the individual evaluations (average value on the top and the respective color of the bar). The more principles that receive a green color, the more environmentally friendly the methodology is.

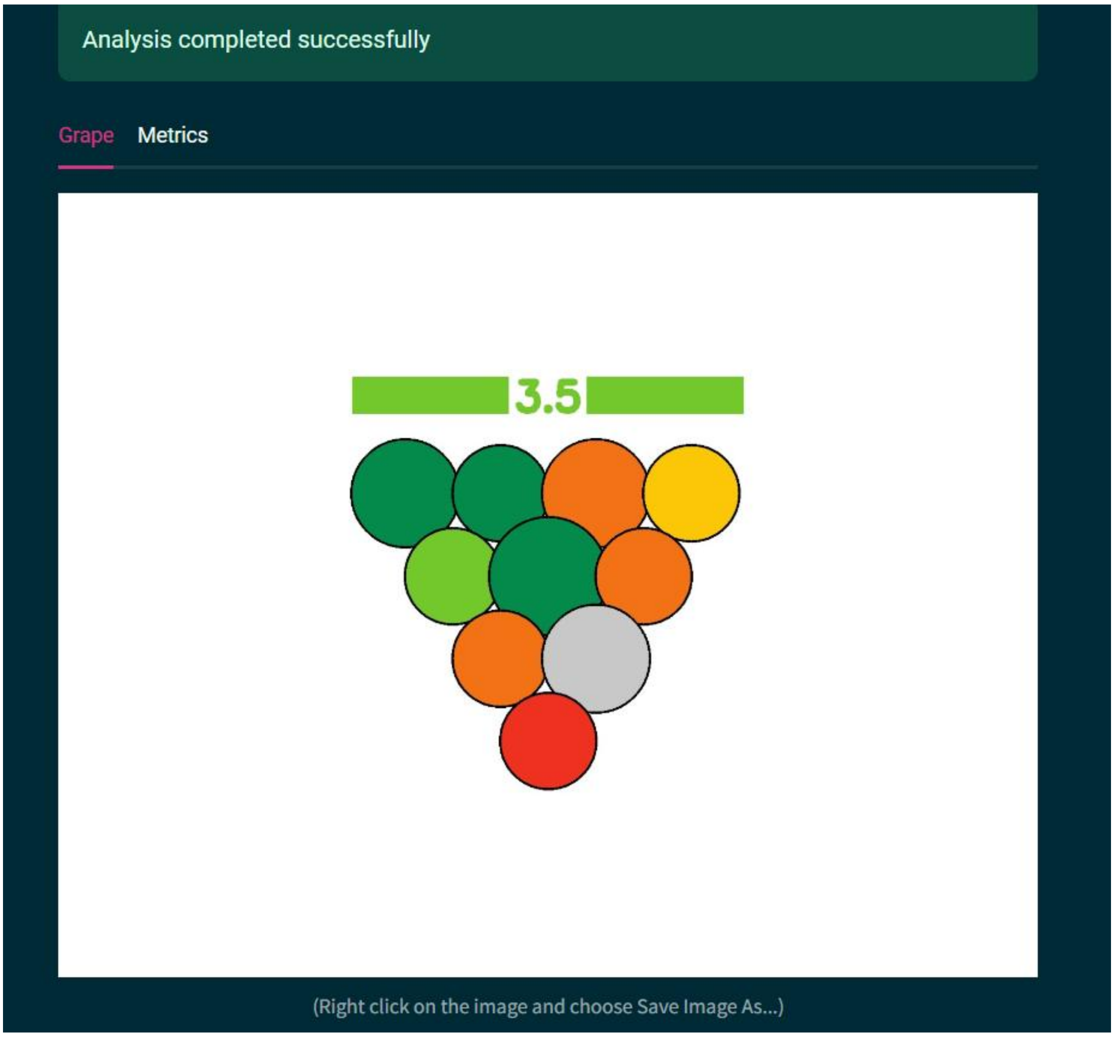

Figure S15: Visual output
